# *Meloidogyne incognita* Fatty Acid- and Retinol- Binding Protein (Mi-FAR-1) Affects Nematode Infection of Plant Roots and the Attachment of *Pasteuria penetrans* Endospores

**DOI:** 10.3389/fmicb.2017.02122

**Published:** 2017-11-01

**Authors:** Victor Phani, Tagginahalli N. Shivakumara, Keith G. Davies, Uma Rao

**Affiliations:** ^1^Division of Nematology, ICAR-Indian Agricultural Research Institute, New Delhi, India; ^2^School of Life and Medical Sciences, University of Hertfordshire, Hatfield, United Kingdom; ^3^Norwegian Institute of Bioeconomy Research, Ås, Norway

**Keywords:** FAR protein, cuticle, *P. penetrans*, endospores, *M. incognita*, host finding, reproduction

## Abstract

Root-knot nematode (RKN) *Meloidogyne incognita* is an economically important pest of crops. *Pasteuria penetrans*, is a nematode hyperparasitic bacterium capable of suppressing the reproduction of RKN and thereby useful for its management. Secreted fatty acid and retinol-binding proteins are unique in nematodes and are engaged in nutrient acquisition, development and reproduction; they are also a component of the nematode cuticle and thought to be involved in the interface between hosts and parasites. Attachment of endospores to the cuticle of second stage juveniles of RKN is the primary step of infection and several factors have been identified to facilitate attachment. In this study, the full length of *Mi-far-1* (573 bp) was cloned from *M. incognita* and characterized. Analysis revealed that the *Mi-far-1* was rich in α-helix structure, contained a predicted consensus casein kinase II phosphorylation site and a glycosylation site. Quantitative PCR showed the highest expression in the fourth stage juveniles and *in situ* hybridization revealed the presence of *Mi-far-1* mRNA in the hypodermis below the cuticle. Single copy insertion pattern of *Mi-far-1* in *M. incognita* genome was detected by Southern blotting. Knockdown of *Mi-far-1* showed significantly increased attachment of *P. penetrans’* endospores on juvenile cuticle surface and also affected host finding, root infection and nematode fecundity.

## Introduction

Plant-parasitic nematodes are important crop pests and have a major impact on global food production system. Around 4,100 species of plant-parasitic nematodes have been identified (Decraemer and Hunt, 2006) and the total crop loss has been estimated to be $173 billion every year (Elling, 2013). Root-knot nematodes (RKN) *Meloidogyne* spp. are widely regarded as the most economically important group as they are polyphagus attacking the majority of crops and inflicting serious yield and quality losses (Jones et al., 2013). Nematicides have been used to control these pests but they are highly toxic to humans, domestic animals and the environment and recently legislation has been used to prohibit their use. Alternative environmentally benign alternatives are currently being sought. Biological control promises to be a good and effective alternative where a suitable biological agent is used to reduce the nematode population density (Hallman et al., 2009; Collange et al., 2011) and is a compatible component of integrated nematode management system.

*Pasteuria penetrans* (Thorne) Sayre and Starr, a Gram-positive, endospore forming nematode parasitic bacterium is a potential candidate to manage RKN and thereby useful as a biocontrol agent. Briefly, endospores adhere to the cuticle of migrating J2s and produce a germ tube, which penetrates the nematode cuticle to establish an infection in the pseudocoelom that proliferates throughout the body of the developing nematode (Davies et al., 2011). The attachment of endospores to the cuticle of second stage juveniles of RKN is the key step in the infection process governed by several factors in which cuticle surface coat plays a pivotal role to facilitate the attachment (Davies and Danks, 1992; Spiegel et al., 1995; Davies and Curtis, 2011).

Nematodes being unable to synthesize fatty acids and retinol, obtain them from their hosts through lipid binding proteins that sustain important life activities (Kennedy, 2000; Fairfax et al., 2009). A series of lipid binding proteins are known to occur in nematodes and based on their molecular weight and structural features, they are classified as either polyprotein allergens/antigens (NPAs),or fatty acid and retinol binding proteins (FARs) (McDermott et al., 1999; Garofalo et al., 2003). FAR proteins are important in cell differentiation, tissue reparation, immune response, energy supply in nematodes and also promote absorption, transportation and specific localization of fatty acids and retinols (Cheng et al., 2013; Iberkleid et al., 2015). The first FAR protein was identified and characterized from *Onchocerca volvulus* (Bradley et al., 2001) and subsequently it has been found to occur in other free-living and animal and plant-parasitic nematodes (Mei et al., 1997; Prior et al., 2001; Basavaraju et al., 2003; Garofalo et al., 2003; Bath et al., 2009; Fairfax et al., 2009; Gabrielsen et al., 2012; Cheng et al., 2013; Iberkleid et al., 2013; Zhang et al., 2015; Vieira et al., 2017).

It is evident that FAR proteins play critical role in development and infection process of plant-parasitic nematodes (Cheng et al., 2013); they inhibit the defense reaction by obstructing the gene expression related to jasmonic acid pathways in host plant (Zhang et al., 2015). The silencing of Mj-FAR-1 in tomato hairy roots, expressing a complementary dsRNA, resulted in decreased infection, while plants with overexpressed FAR protein were found to be highly susceptible to nematode attack (Iberkleid et al., 2013). In animal parasitic species they may also be engaged in modification of local inflammatory and immunological environment of the surrounding host tissues by sequestering pharmacologically active lipids (Garofalo et al., 2003). The role of this protein in the nematode’s own defensive strategies against other microbial pathogens is unknown.

In the present study, a FAR gene was identified from *Meloidogyne incognita* and the full length sequence was amplified and characterized. Knockdown of FAR by RNAi was employed to test its effect on (1) attachment of *P. penetrans* endospores to the cuticle surface of *M. incognita* juveniles and (2) the attraction of gene-silenced encumbered juveniles toward host root. qRT-PCR was used to detect the transcriptional levels in different developmental stages; *in situ* hybridization was performed for localization of mRNA in nematode body and Southern blotting was carried out to find the copy number(s) of the gene in *M. incognita* genome. This is the first study of the role of FAR protein in the attachment process of *P. penetrans* onto *M. incognita* along with its role in pre- and post-infection nematode biology.

## Materials and Methods

### Nematode Population

A pure culture of an Indian isolate of *M. incognita* (Kofoid and White) Chitwood race 1 was multiplied on susceptible tomato plant (*Solanum lycopersicum* L. cv. Pusa ruby) in a greenhouse at ICAR-Indian Agricultural Research Institute, New Delhi, India. Nematode infected tomato roots were washed free of soil and egg masses were hand-picked using a stereozoom binocular microscope. Egg masses were kept for hatching in a double-layered tissue paper supported on a molded sieve of wire gauze in a Petri dish containing distilled water (Whitehead and Hemming, 1965) and freshly hatched juveniles (J2s) were used for experimental purpose.

### RNA Extraction and cDNA Preparation

Total RNA was isolated from about 20,000 freshly hatched J2s of *M. incognita* with TRIzol reagent (Invitrogen, United States) according to manufacturer’s protocol and treated with RQ1 RNase-Free DNase (Promega, United States) to get rid of genomic DNA contamination. The concentration and quality of RNA was assessed with NanoDrop-1000 spectrophotometer (Thermo Fisher Scientific, United States). Complimentary DNA (cDNA) was synthesized from 500 ng of total RNA using cDNA synthesis kit (Superscript VILO, Invitrogen) and stored at -80°C for future use.

### Cloning of Full Length FAR Gene from *M. incognita*

The candidate FAR gene (named *Mi-far-1*) was identified from our transcriptome sequence data of *M. incognita* (unpublished). Accordingly, specific primers were designed (miFARp_F and miFARp_R) (**Table 1**) to amplify the predicted open reading frame (ORF). The cycling conditions include an initial denaturation of 95°C for 5 min, followed by 35 cycles of 95°C for 1 min, 60°C for 1 min and 72°C for 1 min, followed by final elongation at 72°C for 10 min. To clone the full length *Mi-far-1*, primers (miFAR_mf_F and miFAR_mf_R) were designed from the complete cDNA sequence of *M. javanica* retrieved from NCBI database. PCR product was purified and cloned into pGEM-T Easy vector (Promega, United States) and transformed into *Escherichia coli* DH5α competent cells (New England Biolabs). Recombinant plasmids were isolated from the positive clones (QIAGEN Plasmid Miniprep kit) and sequenced.

**Table 1:** List of primers used in this study.

Primer names	Primer sequence (5′–3′)	Product length (bp)	Tm (°C)
miFARp_F	GATTTGGTCCCACCCGAGGT	224	60
miFARp_R	GACCAAGTTGCGGAGCTCGT		
mi_FARq_F	CGAATTGACCGAAGATGACA	106	60
mi_FARq_R	TTCGCTCTTCTCCTTCAATG		
miFAR_mf_F	ATGAGCCGAATAATCCTTTTTGCCGCC	573	60
miFAR_mf_R	TTAGGCTGGTGCAGCAGCGC		
18S_Mi_RT F	TCAACGTGCTTGTCCTACCCTGAA	155	60
18S_Mi_RT R	TGTGTACAAAGGGCAGGGACGTAA		

### Sequence Analysis and Alignment

Sequence homology comparisons was conducted to non-redundant protein database (nr) and non-redundant nucleotide database (nt) using BLASTX and BLASTN^1^. The Coding sequences (CDSs) were predicted by NCBI ORF Finder^2^. Conserved domains were analyzed by Conserved Domain Database in NCBI^3^. The molecular weight, theoretical pI and formula were computed using ProtParam tool^4^. To predict the *N*-glycosylation sites and casein kinase II phosphorylation sites, the NetNGlyc 1.0 Server^5^ and KinasePhos^6^ were used, respectively. Protein secondary structure was predicted using PBIL^7^. Multiple protein sequence alignments and phylogenetic tree analyses were carried out with MEGA6 (Tamura et al., 2013) by neighbor joining algorithm after aligning the protein sequences with ClustalW.

### *In Situ* Hybridization

Gene-specific primers (miFARp_F and miFARp_R) were used for localization of expression of *Mi-far-1* mRNA by performing *in situ* hybridization as described in Kimber et al. (2002). Around 20,000 J2s were concentrated to a 10–15 μl pellet and fixed in 2% paraformaldehyde at 4°C for 18 h, followed by 4 h incubation at room temperature and cut into sections. The gene specific primers miFARp_F and miFARp_R were used to amplify DIG-labeled sense and antisense RNA probes (Roche, Germany) from cDNA of *M. incognita* J2s. DIG-labeled sense or antisense RNA probe was added to the hybridization solution containing the nematode sections, and then rotated at 50°C for 12 h. After hybridization, the nematodes were stained and photographs were taken with Zeiss Axiocam compound microscope.

### Detection of FAR Expression Using Southern Blot

In order to determine the copy number of *Mi-far-1* in *M. incognita* genome, Southern hybridization was performed. Eight micrograms of isolated genomic DNA of *M. incognita* was digested with enzyme *EcoR1* (New England Biolabs), electrophoresed in a 0.8% agarose gel and transferred to nitrocellulose membrane (Bio-Rad Zeta Probe). For probing, the 573 bp fragment of *Mi-far-1* was used. Probe labeling, hybridization and blot development were carried out as described in Papolu et al. (2013) and Dutta et al. (2015) with modification of the hybridization temperature as 42°C.

### Analysis of mRNA Levels at Different Developmental Stages of *M. incognita*

Quantitative real-time reverse transcription PCR (qRT-PCR) was performed to analyze the expression pattern of *Mi-far-1* (Primers: miFARq_F and miFARq_R) in different developmental stages of *M. incognita*. Total RNA was extracted from approximately 1000 pre-parasitic J2s, parasitic J2s, J3s, J4s, young females and egg laying (mature) females and genomic DNA contamination was removed by RQ1 RNase-Free DNase (Promega, United States) treatment. Total RNA (500 ng) was reverse transcribed into cDNA and qRT-PCR was performed in a realplex^2^ thermal cycler (Eppendorf) using SYBR Green Supermix Kit (Eurogentec). Reaction mixture for each sample contained a final volume of 10 μl, comprised of 5 μl of SYBR Green PCR Master mix (Eurogentec), 750 nM of each primer and 1.5 ng of cDNA. To normalize the gene expression level *18S rRNA* (Genbank identifier: HE667742), a constitutively expressed gene was used as the reference. For each sample two biological and three technical replicates were used. The data were analyzed by the ^ΔΔ^*C*t method (Livak and Schmittgen, 2001) and results were expressed as the log2-transformed fold change values and Student’s *t*-test was performed. Primer details are given in **Table 1**.

### Synthesis of dsRNA

The fragment of *Mi-far-1* ORF was cloned into pGEM-T Easy vector and the primer pairs of miFARp_F and miFARp_R were used to amplify the sense and antisense single stranded RNA (ssRNA) products. *In vitro* transcription was carried out with T_7_ and SP_6_ MEGAscript RNAi kit (Ambion, Austin, TX, United States) following manufacturer’s instructions, mixed together in equal concentrations and incubated at 65°C for 10 min followed by 37°C for 30 min. The quantity of dsRNA was determined by NanoDrop-1000 spectrophotometer (Thermo Fisher Scientific, United States) and analyzed by 1% agarose gel electrophoresis and stored at -80°C for future use.

### Knockdown of *Mi-far-1* Using dsRNA

Freshly hatched *M. incognita* J2s (approx. 1000) were collected in a DEPC treated 1.5 ml eppendorf tube and washed thrice with DEPC treated milliQ water. The nematodes were soaked in a solution containing dsRNA for *in vitro* RNAi following the method of Urwin et al. (2002). A concentration of 1000 ng/μl dsRNA was used in 200 μl soaking solution for gene silencing assay and incubated for 24 h in dark on a slowly moving rotator at 28°C. Post incubation, the J2s were washed thrice with DEPC treated milliQ water and total RNA was extracted from the dsRNA treated J2s by NucleoSpin^®^ RNA kit (Macherey-Nagel, Düren, Germany) following manufacturer’s instructions and qPCR was performed to analyze the level of transcript suppression after dsRNA feeding. dsRNA of an unrelated gene (gfp, Genbank identifier: HF675000) was used as non-native negative control and J2s incubated in dsGFP and in soaking buffer without dsRNA were used as the control.

### Assessment of Knockdown Phenotype for *P. penetrans* Endospore Attachment

*Pasteuria* endospores were produced on Adzuki bean with single egg-mass culture of *M. incognita* as described by Rao et al. (2012). To test the effect of dsRNA feeding on attachment of *P. penetrans* (Strain AII-329: *Pasteuria* collection, ICAR-IARI, New Delhi, India) endospores to the J2 cuticle surface, 200 dsRNA-treated J2s were removed from the dsRNA soaking solution and mixed with 100 μl suspension of spores (2.5 × 10^3^ ml^-1^) and centrifuged at 6000 G for 3 min following the method of Hewlett and Dickson (1993). The endospore adhesion was quantified by removing 30 J2s from the suspension after 30 min and spore attachment observed using a Zeiss Axiocam compound microscope and photographed. All assays were performed in triplicate and freshly hatched juveniles were used as control.

### Post RNAi Behavioral and Infection Bioassay

In order to examine the silencing effect of *Mi-far-1* on RKN infectivity together with *P. penetrans*, attraction bioassay was carried out with the dsRNA fed J2s using tomato root in Pluronic gel medium, PF-127 (Wang et al., 2009). Five ml of 23% PF-127 (Sigma–Aldrich) was dispensed into a 35 mm Petri plate and a 5-day-old tomato (cv. Pusa Ruby) seedling was placed in the gel with root tip at the center of the dish. Untreated RKN J2s, dsRNA-treated J2s, endospore-encumbered untreated J2s and endospore-encumbered dsRNA-treated J2s were used for attraction bioassay separately. Approximately 100 juveniles for each treatment were inoculated at 1.5 cm posterior to each root tip and Petri dishes were incubated in dark at 28°C. The attraction of juveniles was studied microscopically and the numbers of juveniles touching the root tip were counted at 2, 4, 6, 8, 10, 12, 14, 16, and 18 h post inoculation. Additionally roots were also stained following NaOCl-Acid fuchsin method (Byrd et al., 1983) to confirm the penetration of juveniles at each time point. The whole experiment contained 10 replicates per treatment and was repeated at least twice.

To test the establishment and reproduction potential of RKN juveniles in the host root upon silencing of *Mi-far-1* (together with *P. penetrans*), CYG growth pouches were used with Adzuki bean [*Vigna angularis* (Willd.) Ohwi and H. Ohashi] as susceptible host. The germinated seeds of Adzuki bean with about 1–2 cm radicals were transferred separately to the CYG growth pouches and maintained in growth chamber at 28°C, 70% relative humidity and 16:8 h light:dark photoperiod. Plants were watered daily with diluted Hoagland’s solution (1:500 stock solution). Untreated RKN J2s, dsRNA-treated J2s, endospore-encumbered untreated J2s and endospore-encumbered dsRNA-treated J2s were used for inoculation with five replications for each treatment as described in Umarao et al. (2013). The points of inoculation were marked on the polythene sheet to keep track of progress of nematode infection and establishment.

### Data Analysis

Bioassay and expression data were analyzed by one-way ANOVA and Tukey’s HSD test (significance level at *P* < 0.05) using SAS version 9.3 for Windows (SAS Software, Inc.).

## Results

### Full-Length FAR Gene from *M. incognita*

**FIGURE 1:**
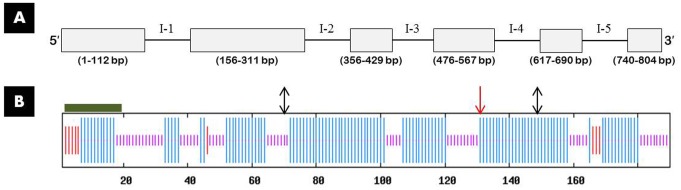
Structure of *Mi-far-1* gene and secondary structure of Mi-FAR-1. **(A)** The structure of *Mi-far-1* was determined by genomic DNA to cDNA from start to stop codon. Exons (rectangles) and introns (solid lines, named I-1 to I-5) were shown with range of exon stretch in bp (numbers below rectangles within parentheses). **(B)** Mi-FAR-1 contained a putative signal peptide at the N terminus (long dark green rod) and was rich in alpha helix (long vertical blue lines) and random coil (short vertical violet lines) structures. Mi-FAR-1 had two predicted casein kinase II phosphorylation sites at amino acids 71 and 150 (double sided black arrow). NetNGlyc 1.0 Server indicated that Mi-FAR-1 has a predicted *N*-glycosylation site (red arrow) at amino acid 131.

According to the EST sequence (224 bp) of FAR gene from cDNA library of *M. incognita*, specific primers for this gene were designed to amplify the 573 bp full-length cDNA sequence and cloned into pGEM-T Easy vector. The plasmid was named as *Mi-far-1* which encodes a deduced 190 bp amino acids. The full-length cDNA sequence was submitted in GenBank (acc. no. MF510388). Based on the cDNA sequences, additional primers were designed to amplify the gDNA of *Mi-far-1*which were 804 bp from ATG to TAA, contained five introns for *Mi-far-1* (**Figure 1A**).

### Sequence Analysis of the Mi-FAR-1 Protein

**FIGURE 2:**
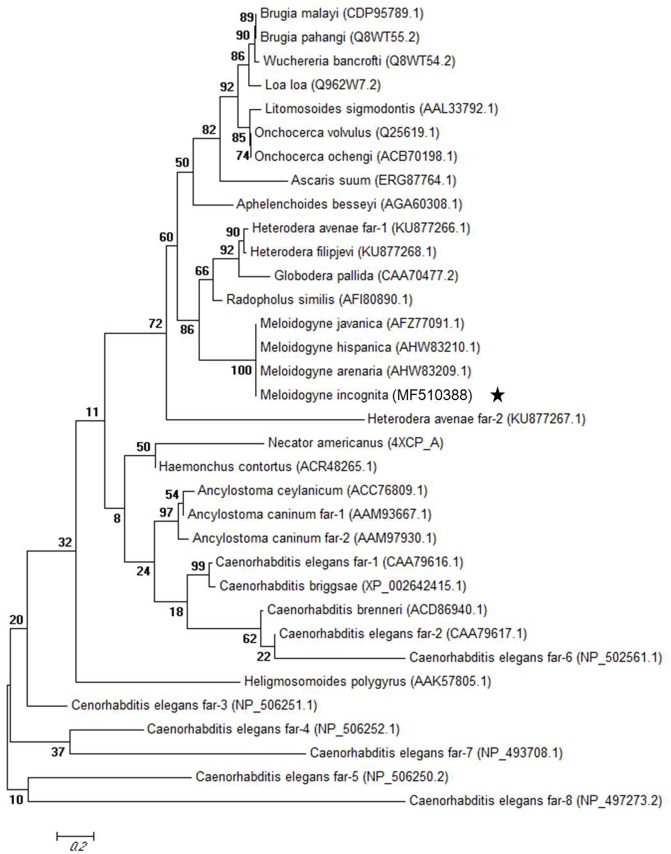
Maximum likelihood phylogenetic tree of 34 fatty-acid and retinol-binding proteins (FAR). The tree for 34 fatty-acid and retinol-binding proteins (FAR) from 16 nematode genera was generated using MEGA6.

The predicted Mi-FAR-1 protein contained 190 amino acids and had calculated molecular mass of 21240.39 Da and predicted molecular formula of C_950_H_1556_N_246_O_297_S_2_ and theoretical pI of 5.34. SignalP and PSORT II Prediction program analyses showed that Mi-FAR-1 possess cleavable hydrophobic secretary signal peptides at the N terminus. Protein secondary structures prediction was done at PBIL revealed that the protein was alpha-helix-rich without beta-sheet. A conserved domain spanning from amino acids 35–174 bp of FAR-1 was identified in *M. incognita*. The results reveal that FAR-1 belongs to the fatty acid- and retinol-binding (FAR) family of proteins. The sequences of *Mi-far-1* and FARs from other nematode species were downloaded from NCBI database and used to conduct homology and phylogeny analyses. Mi-FAR-1 and Mj-FAR-1 (from *M. javanica*) presented the highest homology (98%), and it can be predicted that these two FARs share a similar function (**Figure 1B**). The Maximum likelihood phylogenetic tree constructed with 34 FAR proteins belonging to 16 nematode genera is presented in **Figure 2**. Multiple protein sequence alignments is given in Supplementary Data Sheet 1.

### Molecular Analysis of *Mi-far-1*

**FIGURE 3:**
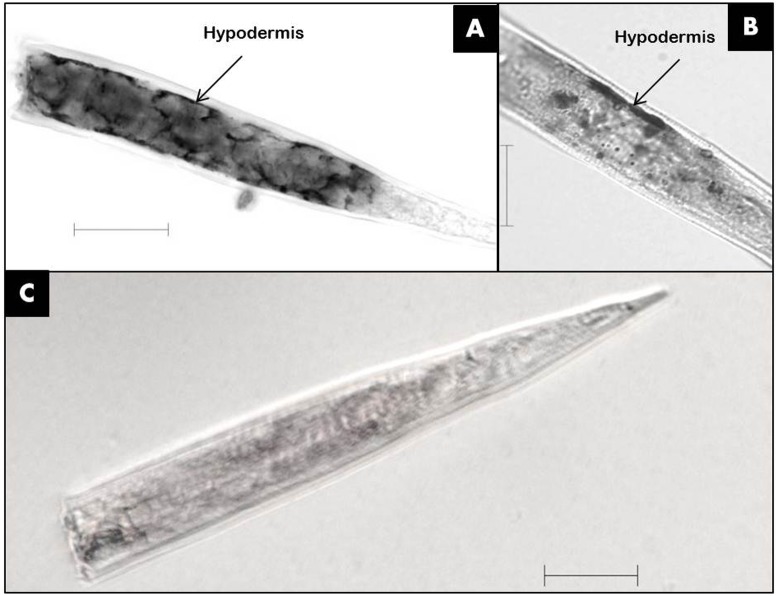
Localization of *Mi-far-1* in pre-J2 of *Meloidogyne incognita* by *in situ* hybridization. **(A,B)** Hybridization to antisense *Mi-far-1* by a DIG-labeled cDNA probe showed the location in the hypodermis; **(C)** hybridization with DIG- labeled sense cDNA probe was used as control. (scale bar: 20 μm).

The mRNA of *Mi-far-1* was localized by *in situ* hybridization in the pre-parasitic J2s of *M. incognita*. The result revealed that the DIG-labeled antisense probes of *Mi-far-1* hybridized in the hypodermis (**Figures 3A,B**) whereas no such signal was detected in the control group with DIG labeled sense probes (**Figure 3C**).

**FIGURE 4:**
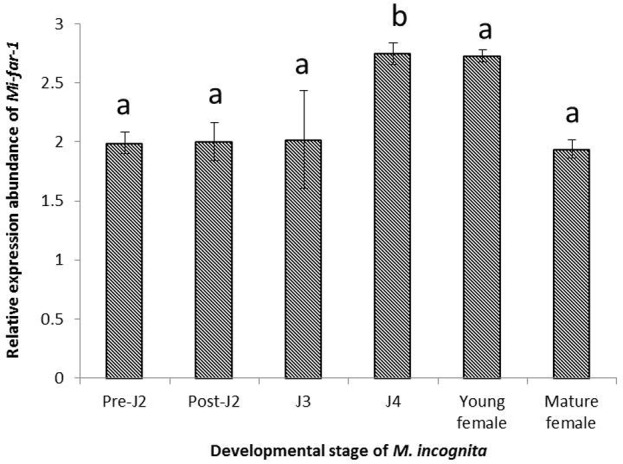
Relative abundance of the transcript of *Mi-far-1* gene in different developmental stages of *M. incognita*. Using 2^-ΔΔCT^ method relative expression level was quantified. Using the transcript level of *Mi-far-1* in eggs as reference, expression of *Mi-far-1* was found to be relatively higher at J4 stage and young females. Each bar represents the mean ± SE and bars with different letters denote a significant difference at *P* < 0.05, Student’s *t*-test.

The results of qRT-PCR showed that *Mi-far-1* was expressed in all developmental stages of *M. incognita*, while highest expression level was detected in J4 stage followed by young females and lowest in egg laying (mature) females (**Figure 4**).

**FIGURE 5:**
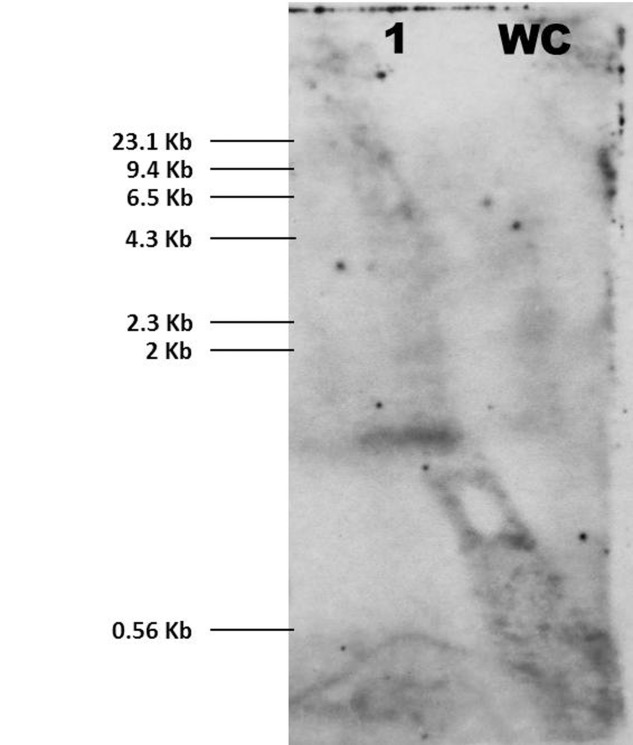
Southern blot for *Mi-far-1*. Probe *Mi-far-1* was used for the hybridization revealed single copy insertion pattern of *Mi-far-1* (1) in *M. incognita* genome. Water control (WC) did not show any hybridization signal.

The result of Southern hybridization indicated that *Mi-far-1* has single copy insertion in the *M. incognita* genome. No hybridization signal was detected in the water control (**Figure 5**).

### Assessment of Knockdown Effect of FAR on *P. penetrans* Endospore Attachment

**FIGURE 6:**
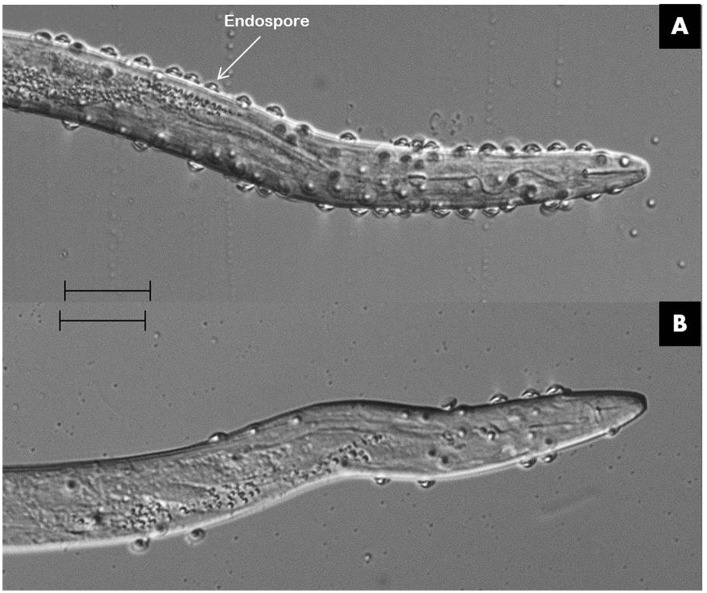
Effect of *Mi-far-1* in attachment of *Pasteuria penetrans* endospores on to *M. incognita*. Silencing of *Mi-far-1* showed increased endospore attachment on to juvenile cuticle of *M. incognita* **(A)** as compared to control **(B)**.

The silencing of *Mi-far-1* upon dsRNA feeding was found to affect the attachment of *P. penetrans* on to *M. incognita* cuticle surface. The average number of endospores attached to the dsRNA treated juveniles was over seven fold higher than the control (285 ± 43 vs. 37 ± 7, respectively; *P* < 0.05) (**Figure 6**).

### Comparative Attraction toward Host and Reproduction of Untreated and FAR-Silenced *M. incognita* with/without *P. penetrans*

**FIGURE 7:**
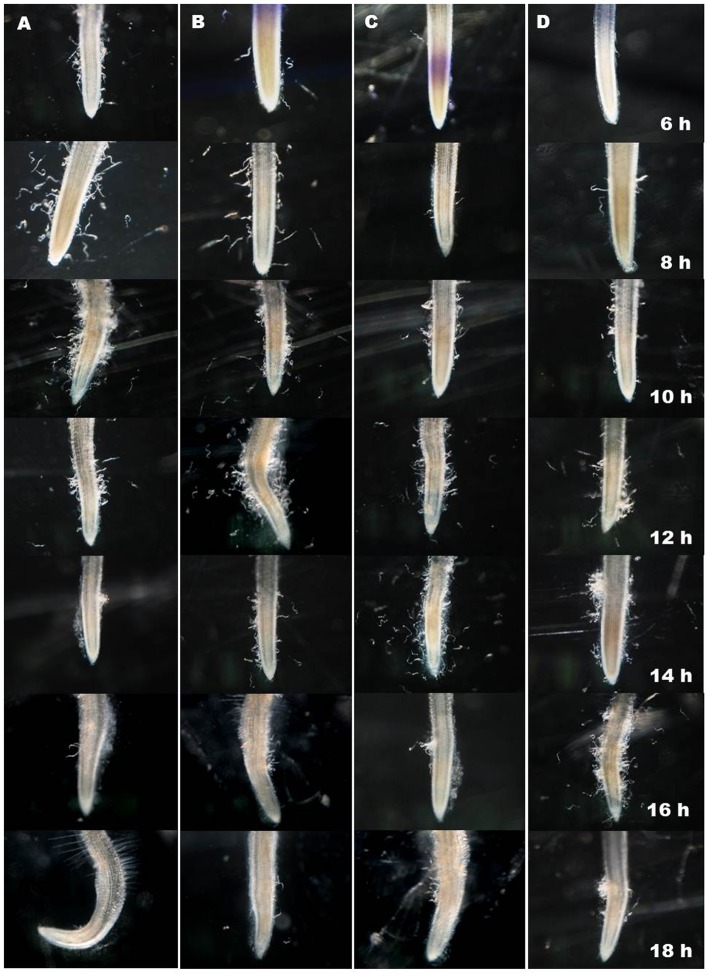
Comparative attraction of *Meloidogyne incognita* toward host root. **(A)** Untreated J2, **(B)** Endospore encumbered J2, **(C)** dsRNA treated J2, **(D)** Endospore encumbered dsRNA treated J2. Maximum J2s around the root tip in **(A–D)** were at 10, 12, 14, and 16 h post inoculation suggesting progressive reduced motility of endospore encumbered, dsRNA fed J2 and endospore encumbered dsRNA fed J2 than control.

The post-RNAi migration of J2s toward tomato root was assessed using attraction assay in PF-127 medium. Silencing of *Mi-far-1* had a negative impact on the host finding ability of *M. incognita* J2s as the worms treated with dsRNA were attracted to tomato root tip in fewer numbers than the untreated worms at 6, 8, 10, 12, and 14 h post inoculation. Maximum number of J2s touching the tomato root tip in control (89.30 ± 2.45) and dsRNA fed worms (79.90 ± 4.28) were observed at 10 and 14 h post inoculation, respectively (**Figure 7**). Further reduction in number of J2s surrounding the root tip suggests that the attracted worms had invaded the host root by that time. The attachment of *P. penetrans* endospores onto juvenile cuticle surface also prolonged the time of host finding for both the untreated and dsRNA treated worms (**Figure 7**). Maximum number of endospore-encumbered untreated J2s (80.20 ± 4.23) were found touching the root tip at 12 h post inoculation, whereas for endospore-encumbered dsRNA-treated J2s, maximum gathering (73.90 ± 4.30) was observed at 16 h post inoculation. The results of penetration study by staining the roots at same time points authenticated the result of attraction experiment (Supplementary Figure 1).

**FIGURE 8:**
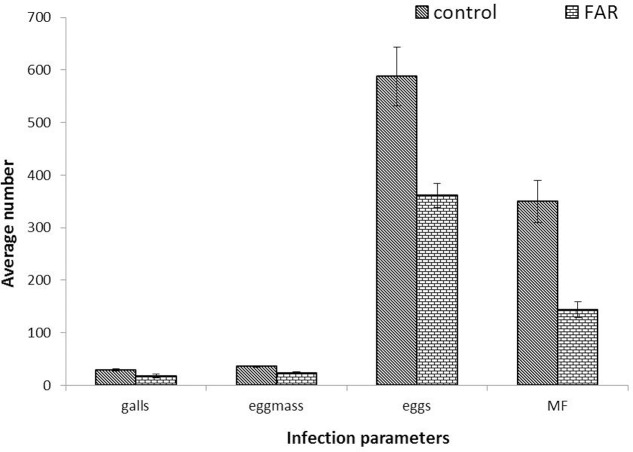
Assessment of parasitism upon silencing of *Mi-far-1*. Number of galls per plant, egg mass per plant, eggs per egg mass and Multiplication factor (MF) was found to be reduced by 58.43 ± 6.42% upon dsRNA treatment over control. (FAR: *Mi-far-1* dsRNA treated).

**FIGURE 9:**
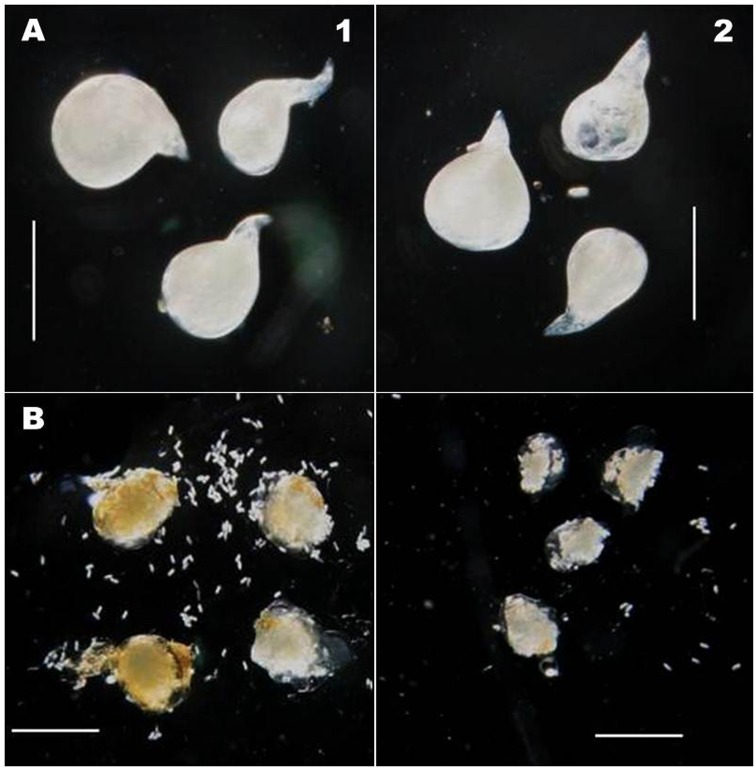
Comparative size of *M. incognita* aaa and eggmass. **(A)** Adult aaa showing no size difference after dsRNA treatment (Length: 619 ± 19.1 μm; Width: 492 ± 13.6 μm) as compared to control (Length: 624 ± 16.3 μm; Width: 495 ± 11.8 μm) (scale bar 0.5 mm); **(B)** dsRNA-treated worms produced relatively smaller egg mass than untreated worms. (scale bar 0.75 mm) (1: Control; 2: dsRNA treated).

**FIGURE 10:**
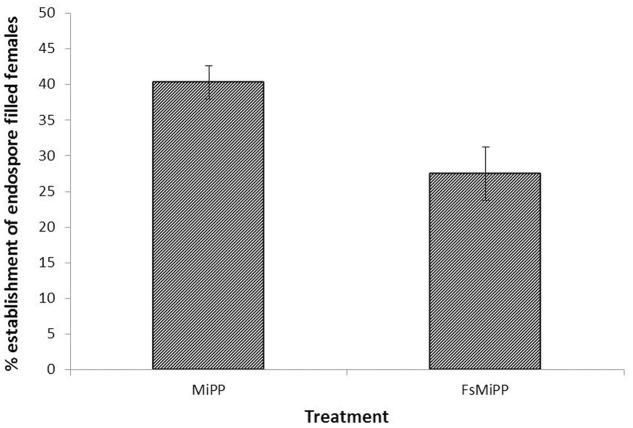
Percent establishment of endospore filled females. Number of endospore filled females were found to be reduced upon dsRNA treatment over control. (MiPP: Endospore encumbered J2; FsMiPP: Endospore encumbered dsRNA treated J2).

The long term effect of *Mi-far-1* silencing on nematode development and reproduction was studied through infection bioassay with treated and untreated nematodes on Adzuki bean in CYG growth pouches. The nematodes treated with *Mi-far-1* dsRNA were found to have lower reproduction as compared to control at 30 dpi. The dsRNA-treated juveniles displayed significant (*P* < 0.05) reduction in parasitism in terms of reduced number of adult females, egg masses, eggs per egg mass and MF (multiplication factor) (**Figure 8**). Average number of galls produced per plant upon infection with dsRNA treated worms was 18.12 ± 3.48, whereas for freshly hatched worms it was 29.12 ± 2.53. The average number of egg masses in control plant (35.62 ± 1.68) was found to be significantly higher (*P* < 0.05) than dsRNA treated case (23.87 ± 2.23). Approximately 588 ± 55.82 eggs were produced per egg mass in control and 361 ± 23.33 eggs in dsRNA treated worms. MF (indicative of reproductive fitness and parasitic success) was reduced by 58.43 ± 6.42% in plants infected with *Mi-far-1* dsRNA-treated J2, as compared to control. The size of egg masses produced by the dsRNA-treated worms were found to be relatively smaller than the control due to a reduced number of eggs being produced, but no significant difference in size of mature females was observed for dsRNA-treated worms over control (**Figure 9**). Hence, silencing of *Mi-far-1* in pre-parasitic J2s retarded the development of the feeding nematodes and eventually reduction in root galling was found in the host plants. Further, the silencing of FAR gene was found to have significant effect on development of *P. penetrans* inside the adult female. Establishment of endospore filled females was reduced by 31.8% in dsRNA-treated worms than the non-treated worms (**Figure 10**).

## Discussion

This study is the first to demonstrate that the FAR protein in *M. incognita* plays a key role in modulating the attachment of *Pasteuria* endospores to the cuticle of infective juveniles and was also found to interfere with host finding, nematode growth, development and fecundity. Present investigation therefore illustrates that FAR protein plays an important role in microbial adhesion to nematode cuticle, and also corroborates earlier studies showing its involvement in other basic biological activities like development, reproduction and also host infection process (Prior et al., 2001; Garofalo et al., 2003; Kuang et al., 2009).

*Mi-far-1* was found to be in the same clade with other plant-parasitic species, viz., *M. javanica, M. arenaria, M. hispanica, Radopholus similis, Globodera pallida, and Heterodera avenae*. It is clear from the phylogenetic analysis that the *Mi-far-1* in *M. incognita* is phylogenetically more distant than the FARs of other animal-parasitic or free-living nematodes. Cheng et al. (2013), in his phylogenetic analysis of FAR proteins from 16 nematode species grouped them as Spirurida, Ascaridida, Tylenchida, Aphelenchida, Strongylida, and Rhabditida whereas, Iberkleid et al. (2013) classified them into five main groups (A–E): FAR proteins from the free-living nematode *Caenorhabditis elegans* (clade A), animal-parasitic nematodes (clade B), root-knot nematodes (clade C), endo migratory plant-parasitic nematodes (clade D) and cyst nematodes (clade E).

*Mi-far-1* was found to be expressed in all the developmental stages indicating its importance throughout the entire nematode lifecycle. The highest expression level of *Mi-far-1* was observed in J4 stage and lowest in the egg laying (mature) females. The expression was found to be progressively increasing in pre-parasitic J2, parasitic J2, J3, and J4; and eventually decreases in the young females followed by egg laying (mature) females. The result is similar to the expression pattern of *Ha-far-1* from *H. avenae* which is highest in the J4 stage and is predicted to play a key role in the pre-reproduction phase of the nematode species (Qiao et al., 2016). In other plant-parasitic nematodes, the highest level of FAR expression in *Aphelenchoides besseyi* occurred in females (Cheng et al., 2013); while the *Mj-far-1* gene in *M. javanica* was highly expressed in the second-stage juveniles (Iberkleid et al., 2015). In animal parasites, the *Hc-far-1* gene expression in *Haemonchus contortus* was higher in adults (Kuang et al., 2009); in *Ancylostoma caninum*, *Ac-far-1* mRNA expression was lowest in males (Basavaraju et al., 2003). In *M. hispanica*, the mRNA of FAR protein was localized in the subventral esophageal glands and possibly secreted into host tissue through the stylet similar to other phyto-parasitic nematode secretory proteins (Duarte et al., 2014). These findings suggest an association between the FAR and the biological characteristics of each developmental stage in root-knot nematodes. Highest expression in pre reproductive J4 stage possibly shows the involvement of the protein in reproduction/egg laying phenomena in *M. incognita*.

*In situ* hybridization results revealed that *Mi-far-1* mRNA was localized in the hypodermal cell layer of *M. incognita*, possibly including the seam cells. The localization of FAR to the hypodermal region of the cuticle is consistent with the findings of other nematode FAR proteins, viz., the *Gp-FAR-1* in *G. pallida* (Prior et al., 2001) and *Ab-FAR-1* in *A. besseyi* (Cheng et al., 2013). The nematode hypodermis is regarded as metabolically very active and plays important roles in communication with the external environment as an interface (Davies and Curtis, 2011). The expression of FAR protein in the hypodermal region may help them to quickly utilize the fatty acids and retinols from the host tissue and to neutralize the plant defense system by compromising with linoleic and linolenic acids and thereby decreasing the jasmonic acid synthesis in the host plant (Zhang et al., 2015).

Increased attachment of *P. penetrans* endospores upon knock down of FAR may suggest that the protein is involved in the formation of a protective coat on the surface of the cuticle and thereby inhibit the endospore attachment. This suggests the possible defensive role of Mi-FAR-1 in *M. incognita* against microbial adhesion. These proteins are predicted to interfere with intercellular lipid signaling to manipulate the host defense reactions. They acquire essential lipids for the parasites and retinol deficiency can alter the character of the host immune response (Garofalo et al., 2003). In animal parasitic species, the FAR proteins have been found to modify the local inflammatory and immunological environment of the host tissues by sequestering pharmacologically active lipids that inhibits the bacterial attachment (Kennedy et al., 1997). In our study, the knockdown of *Mi-far-1* was found to cause chemotactic alteration in the dsRNA-treated worms. The role of FAR protein as an effector for plant responses has already been established in *M. javanica* (Iberkleid et al., 2013). Nematode surface coat is a very dynamic structure and is thought to replace itself within 24 h (Spiegel and McClure, 1995) and in *C. elegans* it clearly has a role in microbial attachment, movement and sexual recognition (Gravato-Nobre et al., 2011).

The reduced number of females and decreased egg production upon *Mi-far-1* dsRNA feeding clearly revealed the role of FAR protein in affecting fecundity. This finding is in accordance with the reduced reproduction of *A. besseyi* upon dsRNA feeding (Cheng et al., 2013) where 48 h following treatment with dsRNA the silencing efficiency of *Ab-far-1* was maximal and the nematode reproduction was lowest. However, treatment with *Mi-far-1* dsRNA for 24, 36, 48, 60, and 72 h consecutively had no effect on enhanced gene silencing with increased exposure time for soaking (data not shown). Since retinol is considered as an essential component for collagen synthesis and embryonic development, down-regulation of *Ab-far-1* by RNAi leads to less binding with retinol causing reduced reproduction in *A. besseyi* (Cheng et al., 2013). Similarly, treatment with *Rs-far-1* dsRNA decreases the reproduction of *R. similis* (Zhang et al., 2015).

The reduced locomotion and accumulation of juveniles at the root surface in the dsRNA fed juveniles may be a result of changes to the biochemical nature of the surface coat which affected the ability of the nematode to acquire traction in its physical environment. This was even more pronounced when dsRNA treated juveniles had a larger number of endospores adhering to their cuticles and were observed to move more slowly. Similar observations were also recorded by Davies et al. (1988) and Vagelas et al. (2012) where the juveniles encumbered with *P. penetrans* were slower than that of healthy juveniles. The spore propagules appeared to interfere with the forward movement of nematode and disorganize the nematode’s labial region turns (Davies, 2009; Vagelas et al., 2012). Nematodes encumbered with extremely high numbers of endospores are likely to have decreased directional movement which may reduce the infective juvenile’s ability to migrate to a host root. This reduced ability has also been enhanced in the dsRNA-treated J2s as compared to untreated J2s. High attachment of endospores onto juvenile cuticle also had an adverse effect on female development. The numbers of infected females in case of endospore-encumbered dsRNA-treated J2s were significantly less than that of endospore-encumbered untreated J2s. Sayre and Wergin (1977) estimated that only 20% of the attached endospores germinate whereas Stirling (1984) indicated that the proportion was nearer to 30%. Heavy number of endospore attachment may exert excess pathogen pressure due to density dependent competition on the juveniles resulting in decreased bacterial pathogenicity and establishment.

The investigations reported here show that the functional role of FAR protein in *M. incognita* is not confined purely to the effects on nematode growth, development and reproduction but also possibly confers immunity against bacterial pathogens and alters the ability of nematodes to move.

## Author Contributions

Conceived and designed the experiments: UR, KD, and VP. Performed the experiments: VP and TS. Analyzed the data: VP and UR. Wrote the paper: VP, UR, and KD.

## Conflict of Interest Statement

The authors declare that the research was conducted in the absence of any commercial or financial relationships that could be construed as a potential conflict of interest.

## Footnotes

1 http://blast.ncbi.nlm.nih.gov/Blast.cgi

2 https://www.ncbi.nlm.nih.gov/orffinder/

3 https://www.ncbi.nlm.nih.gov/Structure/cdd/wrpsb.cgi

4 http://web.expasy.org/protparam/

5 http://www.cbs.dtu.dk/services/NetNGlyc/

6 http://kinasephos.mbc.nctu.edu.tw/

7 https://npsa-prabi.ibcp.fr/cgi-bin/npsa_automat.pl?page=/NPSA/npsa_hnn.html
