# Supplementary material for: Meloidogyne incognita Fatty Acid- and Retinol- Binding Protein (Mi-FAR-1) Affects Nematode Infection of Plant Roots and the Attachment of Pasteuria penetrans Endospores
Source: Front Microbiol. 2017 Nov 1;8:2122. doi: 10.3389/fmicb.2017.02122 (PMC5701614; doi:10.3389/fmicb.2017.02122)
Supplement: Supplementary file 1 [file Presentation_1.PPTX]

## Slide 1
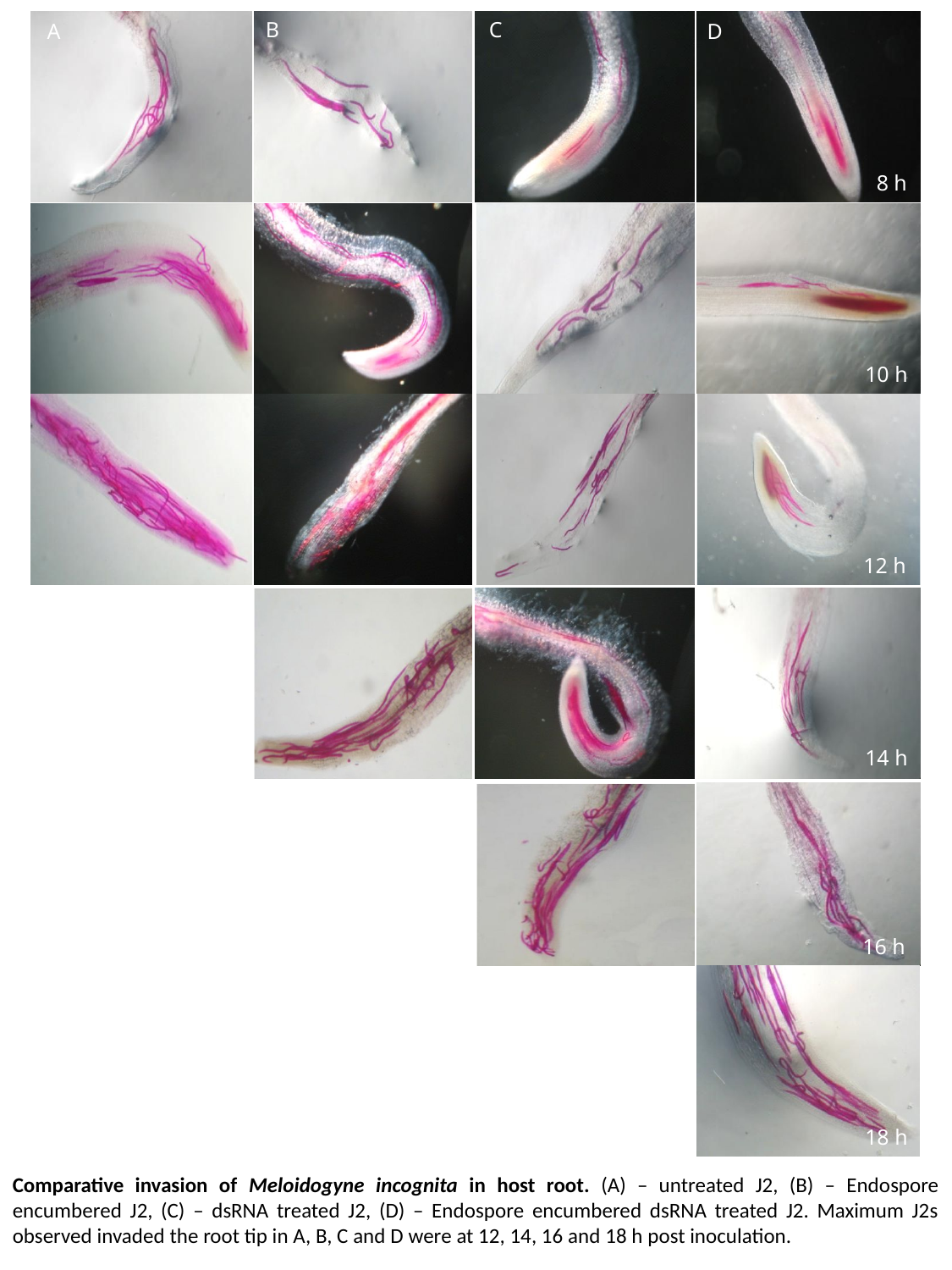

B
C
A
D
8 h
10 h
12 h
14 h
16 h
18 h
Comparative invasion of Meloidogyne incognita in host root. (A) – untreated J2, (B) – Endospore encumbered J2, (C) – dsRNA treated J2, (D) – Endospore encumbered dsRNA treated J2. Maximum J2s observed invaded the root tip in A, B, C and D were at 12, 14, 16 and 18 h post inoculation.

## Slide 2
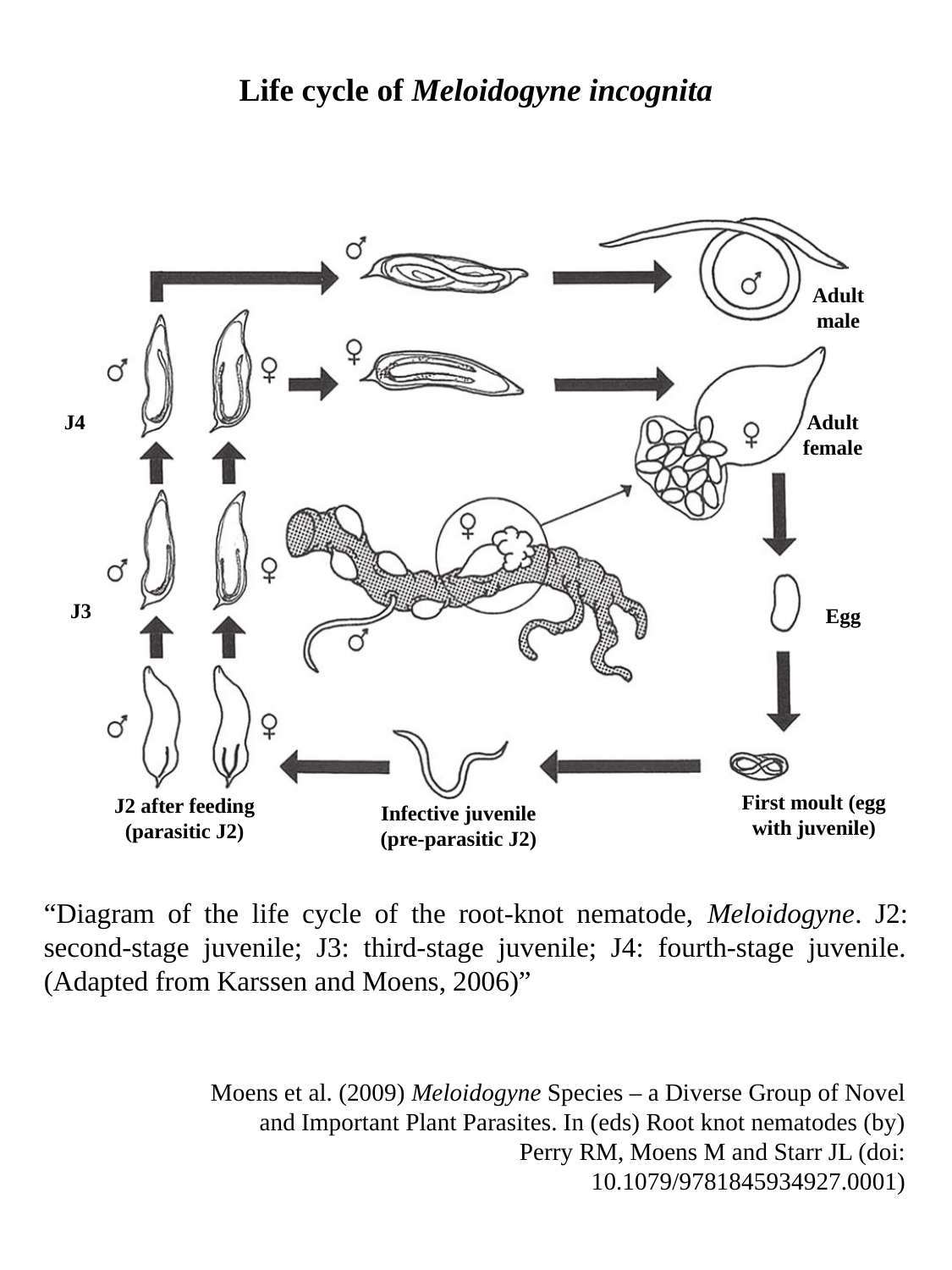

Adult male
J4
Adult female
J3
Egg
First moult (egg with juvenile)
J2 after feeding (parasitic J2)
Infective juvenile (pre-parasitic J2)
Life cycle of Meloidogyne incognita
“Diagram of the life cycle of the root-knot nematode, Meloidogyne. J2: second-stage juvenile; J3: third-stage juvenile; J4: fourth-stage juvenile. (Adapted from Karssen and Moens, 2006)”
Moens et al. (2009) Meloidogyne Species – a Diverse Group of Novel and Important Plant Parasites. In (eds) Root knot nematodes (by) Perry RM, Moens M and Starr JL (doi: 10.1079/9781845934927.0001)
