# Supplementary material for: Meloidogyne incognita Fatty Acid- and Retinol- Binding Protein (Mi-FAR-1) Affects Nematode Infection of Plant Roots and the Attachment of Pasteuria penetrans Endospores
Source: Front Microbiol. 2017 Nov 1;8:2122. doi: 10.3389/fmicb.2017.02122 (PMC5701614; doi:10.3389/fmicb.2017.02122)
Supplement: Supplementary file 2 [file Data_Sheet_1.DOCX]

Cefar3 -----------------MSRLFAFNVFCLVLLRFSAAAPADDSSPFSQILKQHKDLLPSE

Cefar5 ----------------------MLLRFFAILLIFHFSFAADG--IFEAVIESYKDALPPK

Hafar1 -----------------MNAVLSSLIFFGAVLLVSLHAATLPPIDINSIPQEYRELIPTE

Hffar -----------------MNAILSSLIFFGAFLLASLHAATLPPIDINSIPQEYRELIPTE

Gpfar -------------------MQRILLCLTGASFIVLLFGASLPPIDISSIPEQYRELIPKE

Mjfar -----------------MSRIILFAAILAIIAVSTNAASNLPALDMASIPDEYKDLVPPE

Mifar -----------------MSRIILFAAILAIIAVSANGASNLPALDMASIPDEYKDLVPPE

Mhfar ------------------------------------------------------DLVPPE

Mafar ------------------------------------------------------DLVPPE

Rsfar -------------------MRLLSLLAFLIPLFALALSGAIPAIDLNSIPEEYRDLVPPE

Abfar ---------------------MSLRTFVVLFSLFCLAMGATLPLSIQQVPEQLKEVVPEE

Ovfar -----------------------MYHQLILMALIGVIMANVVPFSMSNIPEEYKEFIPEE

Oofar ------------------------------MALIGVIMANVVPFSMSNIPEEYKEFIPEE

Lsfar -----------------------------------------------NIPEEYKEFIPEE

Bmfar MGGERKLKLGNLTAIEKLSLTIKMYHRLILLALVGTTMANVIPFSMSNIPEEYKEFIPEE

Bpfar -----------------------MYHRLILLALVGTTMANVIPFSMNNIPEEYKEFIPEE

Wbfar -----------------------MYHRLILLALIGTTMANVIPFSMSTIPEEYKEFIPEE

Llfar -----------------------MYHQLILLALIGTIMANVIPFSLSNISEEYKEFIPEE

Asfar --------------------MDMLRTAVIASLCIAYSLAFVLPVSMSTIPEEYKEFVPEV

Acfar --------------------MLRLALFAVLFACAFSAPNVEVHK-FEDIPEQYRELIPKE

Acafar1 --------------------MLRLALFAVLFACAFSAPNVEVNK-FEDIPEQYRELIPKE

Acafar2 --------------------MLRLALFAVLFACAFSAPNVEVNK-FEDIPEQYRELIPKE

Hcfar -------------------------------------------S-LEDIPPEYRELIPKE

Nafar ---------------------------XGSSHHHHHHSSGHXFK-YEDIPADYRDLXPPE

Cefar1 -------------------MIRATIILAAVAALAFSAPVPEVPENYDDIPAEYKSLIPAE

Cbrfar -------------------MIRATIVFACLAAVAFSAPIPEVPENYEDIPAEYKSLIPAE

Cefar2 -------------------MIRAFLVVALASVAVFSAPIPEVPQNFDDIPAEYKGLIPAE

Cbfar -------------------------------------PVPEVPENFDDIPSEYKGLIPAE

Cefar6 -------------------MIRIFLVIALASVAVFSAPISRLPQSLDDIPAEFKELIPAK

Hpfar --------------------MLRLGFLALLIVCVCSTP----IKKAEDIPQEVREVLPEN

Cefar4 ---------------------MSKLLLLVLSLLFFITSAFPFGEPQAGGFQKFKNLLPRE

Hafar2 ----MLIMIMPLLFFLLPLFSPFTNAFPAVLTTRTNESDLFRSLPTELDIDTVKEVVPVE

Cefar7 -------------------------------------------MSVASLPECVKNFFPTE

Cefar8 -MFTLRACSLILVSVAVFVLGRPAEEQMTEKDFTNLVFTVEKFDQILKAYSEYKQFMPSY

*

Cefar3 VVQAYQDLSPEEKAALKDVFKNYK----SYKNEGELIAALKEKSSSLGEKAEKLQAKLQK

Cefar5 VVAAFDNLSPGESAIMKEVFMNYD----KFTSIADLIVAIKKKSESLGSFFEKLYIEIDA

Hafar1 VTDFYNTLTEEDKQALKDVAERHE----EFQTEDQAMEALKTKSEKLYNKAVELRNLVKG

Hffar VTDFYNTLTEEDKQALKEVAERHE----EFQTEDQAMEALKAKSEKLYNKAVELRNLVKG

Gpfar VIDFYNTLTAEDKQALKEVAERHE----EFQTEEQAMEALKAKSEKLHSKAVELRNLVKE

Mjfar VTTFYNELTEDDKKILKEVAEKHS----EYATDEDALNALKEKSEKLYTKANELRNLVKD

Mifar VTTFYNELTEDDKKILKEVAEKHS----EYATDEDALNALKEKSEKLYTKANELRNLVKD

Mhfar VTTFYNELTEDDKKILKEVAEKHS----EYATDEDALNALKEKSEKLYTKANELRNLVKD

Mafar VTTFYNELTEDDKKILKEVAEKHS----EYATDEDALNALKEKSEKLYTKANELRNLVKD

Rsfar VTTFYNELTDEDKAVLKEIAGRHE----EFQTEDQALEALKAKSEKLYNKAVELRNLVKG

Abfar VKKFYAELTDEDKSILKEVAANHA----SYENEDQALEALKAKSEKLYNKATELRTLLKT

Ovfar VKNFYKNLTQEDRQILRELASKHA----TFTNEDAALEALKNKSDKLYQKAVELRNFVKA

Oofar VKNFYKNLTQEDRQILRELASKHA----TFTNEDAALEALKNKSDKLYQKAVELRNFVKA

Lsfar VKNFYKNLSIFLCQILRELASKHA----TFTNEDAALEALKNKSDKLYQKAVELRNFVKA

Bmfar VRNFYKDLTVEDKEILRELASKHA----TFANEDAALEALKDKSDKLYKNAVELRNFVKA

Bpfar VRNFYKDLTVEDKEILRELASKHA----TFANEDAALEALKDKSDKLYKNAVELRNFVKA

Wbfar VRNFYKDLTVEDKEILRELASKHA----TFANEDAALEALKAKSDNLYKNAVELRNFVKA

Llfar VRNFYKGLTAEDKEILRDLASKHA----TFANEDAALEALKDKSDKLYKNAVELRNFVKA

Asfar VQNFYKDLTPEDKQILRSIGENHA----QYSSIDAALDALKEKSEKLHAKAVEVRNYIKS

Acfar VADHIKAITEEEKAILKEVLKEYT----KYKDEEEYLAALKQKSPSLHEKAKKFHDFIKA

Acafar1 VADHIKAITEEEKTILKEVLKDYA----KYKDENEYLAALKEKSPSLHEKAKKFHDFIKA

Acafar2 VADHLKAITDDEKKILKEVLKDYA----KYKDENEYLAVLKEKSPSLHEKAKKFHEFIKA

Hcfar AKDFLNGLTDSDKAVLKEIAKDYA----KYKNEDEALAALKEKSPELGAKAEKLHEMVKG

Nafar ARDFLQNLSDGDKTVLKEVFKAG-----PYKNTEESIAALKKKSPELGAKVEKLHAXVKS

Cefar1 VSEHLKSITPEEKAILKEVAKGYK----DFKSEDDFLNALKEKSPTLHEKASKLHQIVKD

Cbrfar VAEHLKSITPEEKAILKDVAKGYK----DFKSEDDFLNALKEKSPALHEKASKLHQIVKD

Cefar2 VAEHLKAITAEEKAALKELAQNHK----EYKTEEEFKAALKEKSPSLYEKAGKLEALLTA

Cbfar VAEHLKAITAEEKAALKELAQKHK----DFKTEDDFKAALKEKSPSLYEKAGKLEALLTA

Cefar6 VTEYLKSITTEEKAAIKEFIKSVMG---GNKSVEELSADIKERSPSLYAKVEKLDVLLRT

Hpfar VVQLILSFTPAEKKVIEEFFNNWE----KFKTEDEALNFFKEKSPSLYAKIENLREILKK

Cefar4 LVEAYSNLSQKDQPDLKDVFRNHQ----NYRNEQEMVNALKMKNPALGARMERRLMALKQ

Hafar2 AADFFDRLTGRDKQVLRQVLAKAD----TYQNTSQVLQDLRNGSSTLYDKAVQVVTSIRA

Cefar7 QLEFSSSITADEKPVLHEVFQKHS----CFSQCGEMIDEVSKKHPELGKRLATVLEGNKK

Cefar8 VLEPLDNITDEQKTQAVQMVNDYHAGKFEPKNYDEYIAIMKKSYPALAGPYETMYNKYKE

:: .. . *

Cefar3 KVDALS-PKPKDFVNELIAGGRGLYARSVNGEKISVSEIKLLIETQVAAYKALPAEAQDE

Cefar5 EIQALT-PETKKFVTEMLGIGRAIYTAQIVGIPLDSKEVLPVFAKQFTSFKSLSDATKEE

Hafar1 KIDALE-PSAKEFVTGMIEKAKAMRPK--TGEKPNLEELRKTANELIEKFKALSDGAKES

Hffar KIDVLE-PSAKEFVTGMIEKAKAMRPK--AGEKPNLEELRKTANELIEKFKALSDGAKES

Gpfar KIDKLV-PGAKTFVTETIEKLKAMRPK--SGEKPNLEELRKGANDTIEKFKALSVEAKES

Mjfar RISKLN-PEAKTFVDTIIEKLKALRPK--KDEKPNLTELRKEANEIVEKFKALSEEAKES

Mifar RISKLN-PEAKTFVDTIIEKLKALRPK--EDEKPNLTELRKEANEIVEKFKALSEEAKES

Mhfar RISKLN-PEAKTFVDTIIEKLKALRPK--KDEKPNLTELRKEANEIVEKFKALSEEAKES

Mafar RISKLN-PEAKTFVDTIIEKLKALRPK--KDEKPNLTELRKEANEIVEKFKALSEEAKES

Rsfar KIDALN-PDAKAFVNAMIEKVKALRPK--PGEKPNLEELRKQANEIIEKYKALSEEAKES

Abfar KIDSLK-PDAKAFVEGIIAKLRALKPK--GEEKPDLKKIREVANEVIDTYKKLAEESKQN

Ovfar KIDSLK-PDAKAFVDEIIAKVRSLRPE--DGQKLDMEKLKQAARDIIAKYEALNEETKEE

Oofar KIDSLK-PDAKAFVDEIIAKVRSLRPE--DGQKLDMEKLKQAARDIIAKYEALNEETKEE

Lsfar KIDSLK-PDAKAFVDEIIAKVRSLRPE--DGQKLDMEKLKQAARDIIAKYEALNEETKEE

Bmfar KIDSLK-PDAKIFVDEIIAKARSLRSD--DGHKLDTEKIKQAARDIIAKYQALSEETKEE

Bpfar KIDSLK-PDAKIFVDEIIAKARSLRSD--DGHKLDTEKIKQAARDIIAKYQALSEETKEE

Wbfar KIDSLK-PDAKTFVDEIIAKARSLRSD--DGHKLDTEKIKQAARDIIAKYQALSEETKEE

Llfar KIDSLK-PDAKAFVDEVIARARSLRSD--DGQKFDTDKIKQAARDIIAKYQALNEETKEE

Asfar KVDSLG-PEAKAFVEEVEAKAKTLRPE--AGHKPDLEKLKTAVREVIEKYKTLPEATKQE

Acfar KVDALG-DEAKAFVKKVIAAARKLHAELLAGNKPSLEELKNTVKKYMAEFEALSAAAKED

Acafar1 KVDALG-DEAKAFVKKVIAAARKLHAELLAGNKPSLEELKNTVKKYVAEFDALTAAAKED

Acafar2 KVDALG-DEPKAFVKKVIAAARKLHAELLAGNKPSLEELKNTVKTYMAEFNALSPAAKED

Hcfar KIDALN-DEAKAFAKEIIAGARKIQAAVVAGNKPNLAELKEKAQKAI-------------

Nafar KIAALG-PEAKGFAEKSIEIARGIKARYYTGNEPTKDDLKASVKEVLKLYKAXSDAGKAD

Cefar1 KVNALN-DEAKAFVKKAIAEGRKIHAQYLAGEKPSLDTLKTTAKTHIEAYKGLSQDAKDS

Cbrfar KVNSLN-DEAKAFVKKAIAEGRKIHAQYLAGEKPSLDTLKATAKTHIEAYKGLSQDAKDS

Cefar2 KFEKLD-ATAQALVKKIIAKGRELHQQYLAGDKPTLDSLKELAKGYIAEYKALSDDAKAT

Cbfar KFEKLD-ASAQALVKKIIAKGRELHQQYLAGEKPSLEELKGLAKGYIEEYKGLSDDAKAT

Cefar6 KLAKLD-PAALALFGKVIAQGLSFRQQFHAGYQPTPEMVKKLFKGYIAEYKTLSENAKAT

Hpfar KVATLS-PESKAFFDKVQSSLKDLHKQILVGDAPSLDMFREVLRKHVDTYKALSADSKKE

Cefar4 KIDGLSSEEAKGFIQNLISTGRQIYAQRLNGQQMDQSQLRQVGMGIAMHYRSLPPYAQQE

Hafar2 IVAGLN-APARTFVDESVAQLR-----NGLGDGVSLSNLKGQAQQLVDRYRRLDASTKEE

Cefar7 RLDGLS-PAAVEYAKKLIHMVTTTLCSLTVGKPID----DADAKRLHQEFQSLSSEDQAA

Cefar8 QVAKLG-PKGQEYCNGLEAQMYTDASPDRVVWACHIFNNAKSAVSGAKALLQDDSE-AAK

. *

Cefar3 LKKNFGGVAKFLEDDKTQTLIAKLLEKNNNQ-----------------------------

Cefar5 LEKTFLGLYKFASNDKIKTEIDKLL-----------------------------------

Hafar1 LKTNFPKISGVIQNEKFQALAKSLLKPAEGAAPAA-------------------------

Hffar LKTNFPKISGVIQNEKFQALAKSLLKPAEGAAPAA-------------------------

Gpfar LKANFPKITGVIQSEKFQALAKSLLKTEGAAPAA--------------------------

Mjfar LKTNFPKITGVIQNEKFQKLAQSLLKPEGAAAPA--------------------------

Mifar LKTNFPKITGVIQNEKFQKLAQSLLKPEGAAAPA--------------------------

Mhfar LKTNFPKITGVIQ-----------------------------------------------

Mafar LKTNFPKITGVIQ-----------------------------------------------

Rsfar LKSNFPKITGVIQNEKFQKLAQSLLKPEATAA----------------------------

Abfar LQETFPQITNVIKNEKFQTLAQGLIKQEN-------------------------------

Ovfar LKATFPNTTKIITNEKFKRIANSFLQKN--------------------------------

Oofar LKTTFPNTTKIITNEKFKRIANSFLQKN--------------------------------

Lsfar LKATFPNTTKIITNEKFKRIANSFLQKN--------------------------------

Bmfar LKVTFPAIAKIIGNEKLKRIASTFLQKN--------------------------------

Bpfar LKVTFPAIAKIIGNEKLKRNASTFLQKN--------------------------------

Wbfar LKVTFPAIAKIIGNEKLKRNASTFLQKN--------------------------------

Llfar LKVTFPPIAKIISNEKLKRVASTFLQKN--------------------------------

Asfar LQTTFPHITKLVKNEKFKKMAKGFLEKNQ-------------------------------

Acfar LKKHFPILTSVFTNEKAKAMIDKHLQN---------------------------------

Acafar1 LKKHFPILTSIFTNEKAKALMDKHLPN---------------------------------

Acafar2 LKKNFPILTSVFTNEKAKALMDKHLQN---------------------------------

Hcfar ------------------------------------------------------------

Nafar FGKQFPFLAKVFESGKAAKFAGEN------------------------------------

Cefar1 IAKEFPILTGFFKNEKVQAMVGQYLN----------------------------------

Cbrfar ISKEFPILTGFFKNEKVQAMVGQYIN----------------------------------

Cefar2 ITAEFPILTGFFQNEKIQAIVGQYVN----------------------------------

Cbfar ITAEFPILTGFFQNEKVQAIVGQYVN----------------------------------

Cefar6 ITDEFPIVVEFFQHEKIQAIIQQIVNY---------------------------------

Hpfar LKKTFPIAARVMSKLVGSN-----------------------------------------

Cefar4 LQSTFPQIFQFMRQMREQRLRSMMGGFFGGGGGIGMGQGMGQGMGMGMGK----------

Hafar2 LRNAFPTVAFVLDNPIIRTMASGLFDIKTNDADNKPRGGGGESARNGKEKNGKRERGGRE

Cefar7 LRKNNPDIKF--------------------------------------------------

Cefar8 IEEAFPEAVKLLNSKKFEAYSIIVNNLKPLDCVKDREQVFNTIKLMDKQSVLTNN-----

Cefar3 ------------------------------------------------------

Cefar5 ------------------------------------------------------

Hafar1 ------------------------------------------------------

Hffar ------------------------------------------------------

Gpfar ------------------------------------------------------

Mjfar ------------------------------------------------------

Mifar ------------------------------------------------------

Mhfar ------------------------------------------------------

Mafar ------------------------------------------------------

Rsfar ------------------------------------------------------

Abfar ------------------------------------------------------

Ovfar ------------------------------------------------------

Oofar ------------------------------------------------------

Lsfar ------------------------------------------------------

Bmfar ------------------------------------------------------

Bpfar ------------------------------------------------------

Wbfar ------------------------------------------------------

Llfar ------------------------------------------------------

Asfar ------------------------------------------------------

Acfar ------------------------------------------------------

Acafar1 ------------------------------------------------------

Acafar2 ------------------------------------------------------

Hcfar ------------------------------------------------------

Nafar ------------------------------------------------------

Cefar1 ------------------------------------------------------

Cbrfar ------------------------------------------------------

Cefar2 ------------------------------------------------------

Cbfar ------------------------------------------------------

Cefar6 ------------------------------------------------------

Hpfar ------------------------------------------------------

Cefar4 ------------------------------------------------------

Hafar2 KDGQNDNKVIEGGKEKDSDRNDKMKWEGKVEGWTMRSVREPNDGILWNGRTNQR

Cefar7 ------------------------------------------------------

Cefar8 ------------------------------------------------------
